# Linking international clinical research with stateless populations to justice in global health

**DOI:** 10.1186/1472-6939-15-49

**Published:** 2014-06-26

**Authors:** Bridget Pratt, Deborah Zion, Khin Maung Lwin, Phaik Yeong Cheah, Francois Nosten, Bebe Loff

**Affiliations:** 1 Department of International Health, Johns Hopkins Bloomberg School of Public Health, 615 North Wolfe St., Baltimore, MD 21205, USA; 2 Victoria University, Melbourne, Australia; 3 Shoklo Malaria Research Unit, Mae Sot, Thailand; 4 Mahidol-Oxford Tropical Medicine Research Unit, Bangkok, Thailand; 5 Centre for Tropical Medicine, Nuffield Department of Clinical Medicine, University of Oxford, Oxford, UK; 6 Michael Kirby Centre for Public Health and Human Rights, Monash University, Melbourne, Australia; 7 Ethox Centre, Oxford University, Oxford, UK

**Keywords:** International clinical research, Global justice, Research ethics, Health capability paradigm, Shoklo malaria research unit

## Abstract

**Background:**

In response to calls to expand the scope of research ethics to address justice in global health, recent scholarship has sought to clarify how external research actors from high-income countries might discharge their obligation to reduce health disparities between and within countries. An ethical framework—‘research for health justice’—was derived from a theory of justice (the health capability paradigm) and specifies how international *clinical* research might contribute to improved health and research capacity in host communities. This paper examines whether and how external funders, sponsors, and researchers can fulfill their obligations under the framework.

**Methods:**

Case study research was undertaken on the Shoklo Malaria Research Unit’s (SMRU) vivax malaria treatment trial, which was performed on the Thai-Myanmar border with Karen and Myanmar refugees and migrants. We conducted nineteen in-depth interviews with trial stakeholders, including investigators, trial participants, community advisory board members, and funder representatives; directly observed at trial sites over a five-week period; and collected trial-related documents for analysis.

**Results:**

The vivax malaria treatment trial drew attention to contextual features that, when present, rendered the ‘research for health justice’ framework’s guidance partially incomplete. These insights allowed us to extend the framework to consider external research actors’ obligations to stateless populations. Data analysis then showed that framework requirements are largely fulfilled in relation to the vivax malaria treatment trial by Wellcome Trust (funder), Oxford University (sponsor), and investigators. At the same time, this study demonstrates that it may be difficult for long-term collaborations to shift the focus of their research agendas in accordance with the changing burden of illness in their host communities and to build the independent research capacity of host populations when working with refugees and migrants. Obstructive factors included the research funding environment and staff turnover due to resettlement or migration.

**Conclusions:**

Our findings show that obligations for selecting research targets, research capacity strengthening, and post-trial benefits that link clinical trials to justice in global health can be upheld by external research actors from high-income countries when working with stateless populations in LMICs. However, meeting certain framework requirements for long-term collaborations may not be entirely feasible.

## Background

International clinical research (ICR) is increasingly being performed in low- and middle-income countries (LMICs), where stark inequalities in health and health research systems may be observed. In recognition of the growing global context of research and lack of benefits accruing to LMICs, Benatar and Singer argued that research ethics should address the linkages between international research and justice in global health [1]. It should serve to connect the enterprise to improvements in health and research capacity in host communities. This call to expand the scope of research ethics has been reiterated by others [2-4].

Scholarship is developing a “broader bioethics agenda of equity and population health” [5] by considering what, if anything, parties from high-income countries owe to parties in LMICs to reduce health disparities between and within countries, and what this means for the conduct of international research [6-10]. Theories of justice drawn from political philosophy provide grounds to establish obligations for actors in rich countries to improve the health of populations in poor countries [11,12]. An ethical framework—the ‘research for health justice’ framework—derived from the health capability paradigm specifies *how* external research actors from high-income countries might discharge their obligation [8]. The health capability paradigm is a theory of justice that extends the work of Sen and Nussbaum and specifically addresses health [11,13]. It was selected as the basis of the ethical framework because it has primary and subsidiary principles that: 1) require the conduct of health research and research capacity strengthening and 2) can establish specific obligations for external research actors from high-income countries to trial participants and host communities [14].

‘Research for health justice’ describes how three aspects of ICR—selection of the research target, research capacity strengthening, and post-trial benefits—should be organized for the enterprise to advance the ends of global justice. The framework recognizes that actors beyond researchers (i.e., governments, research funders, and sponsors) have obligations of justice and that the fulfillment of their obligations is necessary for researchers to satisfy *their* obligations. Its requirements differ from the justice requirements articulated in international research ethics guidelines such as responsiveness and reasonable availability [8]. This reflects the fact that the framework and international guidelines are intended to address different objectives—namely, justice at the macro-level (reduction of global health inequities) and justice at the micro-level (distributive justice in the context of single trials). Linking international research to global justice entails different ethical requirements than achieving a fair balance of benefits and burdens in individual trials does.

‘Research for health justice’ requires that ICR be undertaken in communities whose health status is far from the optimal global level. The responsiveness requirement, however, places no restrictions on the selection of host communities [8]. It allows international research to be performed in high-income countries or with populations that have decent health in LMICs. Under ‘research for health justice’, ICR must address health conditions that are major contributors to the poor health status of worst-off^a^ communities in LMICs. It is not sufficient for the condition-under-study simply to be prevalent or represented in the host community (i.e., to be a health need), which is permitted by international guidelines, including the *Declaration of Helsinki* and the CIOMS guidelines [8]. Resultant interventions must be culturally appropriate and likely to be made available to host communities post-trial. ICR should also be conducted as part of a long-term collaboration with local researchers and institutions. Over time, these collaborations’ disease focus should shift to reflect any changes in the disease burden of their host communities. They should build local researchers and institutions’ capacity to independently conduct clinical research on health conditions that are primarily responsible for their population’s poor health status. International guidelines neither require researchers and sponsors to engage in (long-term) collaborations nor offer guidance on the nature of the disease focus of these collaborations’ research. They largely do not clarify the nature of research capacity to be strengthened or call for building capacity beyond the duration of a single trial [8].

‘Research for health justice’ requires that post-trial access to efficacious interventions be supported in host communities by a global health institution and, wherever possible, be delivered by state health systems [8]. Obligations are allocated according to the health capability paradigm’s functional requirements principle, so parties are assigned obligations because the functions they typically assume make them particularly capable of fulfilling the obligations [11]. As a result, in contrast to international guidelines, researchers and sponsors are not given the *primary* obligation to provide interventions proven efficacious post-trial^b^. That responsibility is allocated to global health institutions that work towards creating access to medicines [8]. (A more comprehensive summary of framework requirements is provided in Additional file 1 and Additional file 2.)

Although research actors’ obligations of global justice are starting to be defined, practices capable of fulfilling them have not been described. As has been noted,

*[c]urrently, little information is available to ascertain what types of research are actually being undertaken in developing countries, how much work is being done, the benefits that studies presently offer communities, and whether research addresses the needs of developing countries, developed countries, or both *[15].

Few studies have examined compliance with ethical requirements relating to justice irrespective of how it is conceived (i.e., at the micro or macro-level). In 2001, a study exploring the extent to which international research ethics guidelines were observed solicited researchers’ perspectives on their projects’ responsiveness to local needs, standard of care for participants, provision of study interventions post-trial, and capacity-building [16]. More recently, the Global Campaign for Microbicides conducted a study assessing the standard of care achieved in six African microbicide trials [17]. These studies did not investigate *how* ethical requirements relating to justice at the micro-level were fulfilled. Yet that information is essential to translating ethical guidance into practice. Fulfilling justice requirements in resource-poor settings is complex and empirical research can provide critical information for research actors [18].

This paper explores how ethical obligations connecting international research to justice in global health can be achieved when conducting research with stateless populations^c^. To test whether and how ‘research for health justice’ can be upheld, we undertook case study research with the Shoklo Malaria Research Unit (SMRU). Its ongoing vivax malaria treatment (VHX) trial was examined for consistency with framework requirements. Data collected through in-depth interviews with trial stakeholders, direct observation at trial sites, and examination of trial-related documents were analyzed according to the principles of thematic analysis. During this analysis, shortcomings of the framework were identified, as the VHX trial drew attention to contextual features that, when present, rendered its guidance incomplete. Insights from this case study allowed us to extend ‘research for health justice’ in ways not previously contemplated. Once gaps in the framework were addressed, we were able to complete our assessment of the VHX trial. Even though this was a retrospective application of a new framework^d^, we found that Wellcome Trust (funder), Oxford University (sponsor), and trial investigators upheld most of their obligations. We describe the facilitating and obstructive factors. Where stakeholders did not uphold obligations, plausible reforms to practice are suggested.

Some of the data generated by our empirical research on SMRU are mentioned in an earlier methods paper [18]. They were included at the request of reviewers in order to demonstrate the outputs of our research method. In this paper, the data are analyzed using ‘research for health justice’ as the normative framework because the aim of our case study research was to investigate whether and how SMRU’s VHX trial was able to promote global health justice. We, therefore, assessed the practices of the VHX trial’s funders, sponsors, and researchers for alignment with a framework that describes how ICR can advance such ends (rather than with ethical requirements designed to promote justice at the micro-level). (The methods paper briefly evaluates *researchers’* adherence to international guidelines’ requirements for responsiveness, research capacity strengthening, and reasonable availability.)

## Methods

Case study methodology was used in this study because it enables exploration of how and why a complex social phenomena works and can bring out important contextual features [19]. We describe the case under study, the specific methods used to collect data on it, and the role of SMRU co-authors in the study below.

### The case under study

We selected SMRU and its VHX trial as our case study because, for the past 25 years, SMRU has been consciously designing its clinical trials to meet the health needs of its host community. It was, therefore, more likely that an SMRU trial would demonstrate whether ‘research for health justice’ requirements could be upheld than many other clinical studies and provide data on how this was achieved.

SMRU is a field research site of the Mahidol-Oxford Tropical Medicine Research Unit located in Mae Sot, Thailand. It has been conducting operational research since 1985 with Karen and Myanmar refugees, migrants, and displaced persons living on the Thai-Myanmar border. The Karen, one of the largest ethnic groups in Myanmar and northern Thailand, has been engaged in armed rebellion against the Myanmar military forces since 1949. This conflict has forced hundreds of thousands of refugees to flee to Thailand. Economic stagnation in Myanmar has also led millions of migrant workers to settle in the border region and seek work in Thailand. As such, the border population is not homogenous. Refugees are primarily Karen and physically situated in Thailand in camps like Mae La. They may stay in these camps for a few months to decades. The Thai government considers these refugees to be under its authority [20]. Migrants live on either side of the border. Those in Myanmar may routinely cross into Thailand for work or to access health care at an SMRU clinic. Migrants are more mobile than the refugees based in camps. Some of those entering Thailand for work are registered migrants with work permits. In 2004, there were 610,106 registered migrants and 1.3 million illegal migrants [20]. A proportion of the border population are effectively permanent residents of Thailand while others regularly cross the border^e^.

Although Thai and Myanmar hospitals are in the vicinity of the border, they are physically and financially difficult for refugees, illegal migrants, and displaced persons to access. Reaching a Thai hospital requires travelling at least 15–20 kilometers beyond the border and/or passing through military checkpoints intended to prevent Myanmar nationals from crossing into Thailand. As neither Myanmar nor Thailand make their health systems accessible to the border population, over the past fifteen years, SMRU has established free clinics to fill the health care gap. It is both a research unit and health care provider. SMRU’s research profile includes falciparum and vivax malaria, respiratory diseases, malaria in pregnancy, and child health. There may be five to ten ongoing clinical trials at any time.

The VHX trial is funded as part of a Wellcome Trust Programme Grant. It seeks to describe the epidemiology and compare the efficacy of three treatments for vivax malaria—chloroquine/primaquine, chloroquine, and artesunate (web reference: http://clinicaltrials.gov/ct2/show/NCT01074905). Trial sites are five SMRU clinics—Mae La, Wang Pha, Mawker Thai, Mun Ru Chai, and Mae Kon Ken—and there were roughly 410 VHX trial participants at the time of our research. Of the five clinics, only Mae La Clinic is located in a refugee camp. The four remaining clinics primarily service Myanmar migrants and are located just over the Thai side of the border. Myanmar nationals do not need to cross a Thai military checkpoint to reach these clinics. However, habitual flare-ups of violence along the border can present a barrier to accessing SMRU clinics. As SMRU has been operating for over 25 years, the VHX trial is performed as part of a longstanding research collaboration.

### Case study methods

Data on the VHX trial were collected using a triangulation approach that relied on in-depth interviews, direct observation, and document analysis. Nineteen semi-structured in-depth interviews were conducted with four types of VHX trial stakeholders—investigators (five interviews), Tak Province Border Community Ethics Advisory Board (T-CAB) members (four interviews), trial participants (eight interviews), and Wellcome Trust science portfolio advisors (two interviews). A series of open-ended questions was designed such that interviewees were asked to describe, first, their roles and responsibilities during each stage of the VHX trial and, second, their perspective on the health impact of the trial on participants and the border population [18]. For interviews with trial participants and T-CAB members, a translator was used. Interviews were an average duration of 72 minutes [18].

Interview data were supplemented by direct observation at four of the five VHX trial sites over a five-week period in March and April 2011 and by an examination of trial-related documents. To collect data, BP travelled to SMRU clinics nearly every weekday over a five-week period. The main observation strategy was to try and identify VHX trial participants based on the medical tests they received, to confirm this with SMRU medics, and then to continue observing those individuals to determine what trial-related and ancillary care they received. BP also observed the research skills of the Karen clinic staff [18]. Participant recruitment and data collection methods are comprehensively described in Pratt et al. (2012) [18]. Ethical approval for the study was obtained from the Ethics Committee of the Faculty of Tropical Medicine at Mahidol University, the Tropical Research Ethics Committee at Oxford University, and the Monash University Human Research Ethics Committee.

Written consent was obtained from all interviewees by either BP, KML (with BP also present), or a translator (with BP also present). Prospective interviewees were provided with both a Participant Information Sheet and Consent Form in English or Burmese. Prior to signing the Consent Form, interviewees had the research project explained to them, read the Participant Information Sheet and Consent Form (or had the forms read to them if they could not read), and were given time to consider entering the study and to ask questions. The Participant Information Sheet and Consent Form used for the case study research detailed the aims, methods, anticipated benefits and risks of the research, data storage procedures, study investigator contact information, and complaints procedure. These documents informed prospective interviewees that taking part in the study was voluntary, that they could choose not to participate in the study, and that they could withdraw at any stage of the study, without giving a reason. Prospective interviewees were also informed that their names and responses would not be shared with anyone outside the study team, that the information they gave during interviews would be de-identified, and that their names would not be used in any reports or publications based on the study.

Participants who chose to take part in the study and who could read and write signed two copies of the Consent Form, one for their records and one for the study team’s records. (They were given a copy of the Participant Information Sheet to keep as well.) Participants who were not literate were also asked to sign two copies of the Consent Form. This was because, despite not being able to read or write, illiterate individuals were able to sign their name and it was considered insulting to ask them to provide a thumbprint instead of a signature. In such cases, the person who took their consent signed each copy of the Consent Form, affirming that s/he “hereby testifies that the content of this letter of consent and the explanation about the study were read to the participant. The participant had the opportunity to ask questions about it and any questions that have been asked have been answered to his/her satisfaction. He/she is pleased to willingly participate in this study voluntarily.”

Interviews were transcribed verbatim and translated from Burmese to English where required. To ensure the accuracy of translation, two interviews were re-translated by a co-investigator who is fluent in Burmese and English. There were no significant discrepancies between his translation and that of our transcriber. Data were analyzed according to the principles of thematic analysis described in Braun and Clarke [21], with co-coding performed independently by two researchers. Once themes were identified that pertained to the selection of the research target, research capacity-building, and post-trial benefits, we assessed whether their collated data extracts provided evidence that the VHX trial met ‘research for health justice’ framework requirements. The results of that analysis are subsequently discussed, following clarification of the framework’s guidance on obligations to stateless populations and post-trial benefits in operational clinical research.

### Role of SMRU authors

Three of the authors of this paper are employed by SMRU. In this section, we describe who they are and their role in the data collection phase of this research study. We consider how their role in collecting data might have affected what data was generated and whether their involvement might limit the objectivity of this study.

KML is a physician-investigator from Myanmar who has worked at SMRU for over five years and is based in Mae Sot. PYC is the Head of the Clinical Trials Support Group in the Mahidol-Oxford Tropical Medicine Research Unit. She is primarily based in Bangkok but travels regularly to Mae Sot. FN has been the director of SMRU since its inception in 1985 and is based in Mae Sot.

These authors were involved in the development of the interview question guides for T-CAB members and trial participants. This ensured that the questions were relevant to the nature of T-CAB members and trial participants’ involvement in the VHX trial and would be understood by such individuals. In March 2011, BP consulted with FN, KML, PYC, and a VHX trial investigator regarding the content of the interview guides. Changes were made to the T-CAB member and trial participant interview guides as a result. The aforementioned individuals advised that trial participants and T-CAB members would be better able to respond to targeted rather than open-ended questions. In order to elicit as much narrative as possible and to avoid getting only yes or no answers, BP added the phrases “Why” or “Please provide an example” to the end of certain questions. These individuals also noted that asking participants about what will happen when the VHX trial is over (i.e. in the future) might not be well understood or easily translated into the Burmese language. They recommended that the term “malaria” rather than vivax be used because trial participants would not know that there are different forms of malaria. Together, the VHX trial investigator, KML, and BP re-wrote the trial participant interview guide, *maintaining the integrity of its content while altering some of its wording*. BP then re-wrote the T-CAB interview guide and KML approved the new version.

Based on a clear criterion, FN, PYC, and KML also assisted in the identification of suitable T-CAB members to interview. Five of fourteen T-CAB members were selected because they lived in the villages near the VHX trial sites and would, therefore, be best able to describe the impact of the trial on its host communities. Four of those T-CAB members were available and consented to be interviewed. The fifth T-CAB member was in Myanmar at the time of this study and was not able to be contacted for interview.

KML was responsible for recruiting and interviewing T-CAB members in Burmese. During these interviews, BP was present and KML repeated interviewees’ responses in English to her after they answered each question in the interview guide. T-CAB members have an ongoing relationship with KML. KML and PYC facilitated the establishment of the SMRU T-CAB and act as its coordinators, facilitating monthly meetings where SMRU investigators present on upcoming clinical research projects. Although KML and PYC attend and facilitate each T-CAB meeting, they do not participate in T-CAB members’ deliberations. Even so, T-CAB members may perceive them to be authority figures. Perhaps this impacted their decision to consent to be interviewed as part of this study, but we would emphasise that T-CAB members were not pressured to participate. Their recruitment proceeded in an ethical manner and they understood that their decision to participate had no implications for their involvement in T-CAB or their future treatment. (T-CAB membership is voluntary, with members receiving compensation for their time and transportation costs. Members also report benefits for themselves through their involvement such as enhanced knowledge and roles as educators in their communities^f^).

As part of the interviews with T-CAB members, KML asked questions to determine what the health concerns were in their communities and whether vivax malaria was one of them. He also asked T-CAB members to describe how SMRU research improves health in their communities during and after trials and how SMRU research benefits their communities. These questions were pertinent to assessing whether the VHX trial was consistent with ‘research for health justice’ requirements for selecting research targets and post-study benefits. We acknowledge that having KML conduct these interviews had potential to affect interviewees’ responses, perhaps generating an overly favourable response about the impact of SMRU and/or an agreement that vivax was a major health concern. T-CAB members stated that once SMRU research indicates that new treatments are more effective than those currently in use in its clinics, SMRU doctors will revise their practices and use the new medicines. This does not appear to be an exaggerated assessment of SMRU’s impact. It is consistent with comments made by VHX trial investigators in their interviews and with SMRU’s practices relating to its previous research on falciparum malaria treatment. T-CAB members understand the difference between vivax and falciparum and were actually more likely to cite falciparum as a concern than vivax. They were also willing and able to identify non-malarial health concerns when asked.

Finally, KML was responsible for re-translating two T-CAB interview transcripts in order for a comparison to be made with the translation of the same transcripts by our Melbourne-based transcriber. This was done to assess the accuracy of the transcription done by our transcriber. There were no significant differences between KML’s translation and that of our transcriber.

KML, PYC, and FN were not involved in any of the other interviews with VHX trial participants, investigators, or funder representatives. They were also not involved in the direct observation aspects of the study. We believe that the nature of the involvement of these three authors was not of a kind to raise concerns about the validity of study findings.

## Extending the ‘research for health justice’ framework

The ‘research for health justice’ framework made two assumptions about ICR that were not reflected in the VHX trial: 1) all individuals are citizens of a state and 2) ICR is directed towards evaluating new health interventions. The Karen and Myanmar border population is essentially stateless and the VHX trial aims to optimize an existing treatment for vivax malaria. To apply the framework to the VHX trial, it was necessary to develop guidance on what is owed to stateless populations and the content of post-trial benefits in operational clinical research. Extending the framework did not force a deviation from health capability paradigm principles.

### Obligations to stateless research populations

The ‘research for health justice’ framework did not identify who external institutions and researchers should work with when trial participants are stateless. Are external VHX trial stakeholders expected to work with research institutions and researchers in Myanmar to build their clinical research capacity and rely on the Myanmar health system to create post-trial access to study interventions? Or are they required to work with Thai research institutions and researchers and rely on the Thai health system?

The fact that individuals have fled Myanmar does not absolve the state of responsibility for fostering their health capabilities. Under the health capability paradigm, states have the primary obligation to ensure their populations’ freedom to be healthy. Where states are unable or unwilling to provide their populations’ health entitlements, the paradigm obliges global actors to help states meet their obligations. Myanmar has an obligation to reform its regime such that it is better able to meet the (health) needs of its ethnic minorities. Nonetheless, when citizens of a state seek refuge in another state, we take the position that the state of refuge assumes secondary responsibility for achieving just health outcomes for those individuals, provided it is capable. Global actors and institutions have an obligation to assist the state of refuge to promote the health capabilities of the stateless population. Accordingly, external research actors have an obligation to build research capacity at the institutional level in the state of refuge and at the individual level in the state of refuge and among the migrant and refugee populations^g^.

In allocating obligations of justice to the state of refuge, the framework assumes that states that are able to meet their obligations to refugees will do so. In this instance, the assumption is that Thailand will take responsibility for the stateless populations within its borders, giving global actors clear partners with whom to work. Again, however, the context of the VHX trial is inconsistent with framework assumptions. Thailand’s health system is generally inaccessible to the border population, though health care is available to some registered migrants^h^. Where states do not uphold their obligations and post-trial access cannot be coordinated through state health structures, external actors (including global health institutions), nonetheless, retain their obligations under the framework.

### Post-trial benefits in operational clinical research

The ‘research for health justice’ framework establishes an obligation to facilitate sustainable access to new interventions in host communities of phase II, III, and IV clinical trials. Its guidance could not be applied to the VHX trial because it is *operational* clinical research designed to change treatment practice so an existing intervention is utilized according to a newly optimized regimen. For operational research, the obligation to provide post-trial benefits is better articulated as an obligation to change treatment practice. Changes to practice will ideally be made accessible to a research population through its state’s health system.

## Results and discussion: Alignment of SMRU’S VHX trial with ‘research for health justice’

### Selection of the research target

For SMRU’s VHX trial to meet the framework’s criteria for selecting a research target, its host community must exhibit a large gap in health status from the optimal global level. Vivax malaria should be a major contributor to that gap in health status. A need should exist for clinical research on vivax in the host community and primaquine must be appropriate for use by trial participants and their communities. As a longstanding research collaboration, SMRU is also required to demonstrate that the content of its research agenda has changed in accordance with any changes that might have taken place in its host community’s burden of disease over time [8].

#### ***Selection of the host community***

Francois Nosten came to the Shoklo camp in 1985 to establish a Médecins Sans Frontières clinic for Karen refugees. He and Nick White subsequently created SMRU in order to do clinical research with this population, as there was a need to improve malaria treatment. Resistance was emerging in Shoklo camp to then-current interventions. Maternal mortality was high, with the estimated maternal mortality rate at 499 per 100,000 live births [22].

SMRU later expanded its research to migrant workers and displaced persons outside refugee camps like Shoklo. In part, this was because malaria transmission dropped in the camps but remained high in surrounding rural areas. The estimated maternal mortality rate in the migrant population was 588 per 100,000 live births in 1996–2000 [22]. By expanding its research population, SMRU demonstrates how a research group can evolve so that it continues to address the health needs of the worst-off. Investigator 02 suggests that the choice to expand was also made because the migrant population was at high-risk for emerging drug resistance. He states,

they are unprotected population because the population along the border, most of them, they are not recognized by any of the country. So they are not recognized by Thailand and they are not recognized by Myanmar. They are not under any medical cover for the health care system of the neighbor countries and another one is the their migration pattern is very quick, so that means that the transmission of the disease pattern is very quick and then the transmission of the drug resistance is very quick… so we need to know the treatment we are using is effective and especially the malaria treatment. We need to know, are we using the right treatment?

Due to their mobility and poor access to care, migrants and displaced persons were at high-risk of acquiring diseases and promoting the emergence of drug resistance to existing treatments. This meant the population continually required revised treatments for diseases like malaria.

The selection and expansion of SMRU’s research population was consistent with ‘research for health justice’, as it was based on a combination of the border population’s poor health and need for clinical research. Today, the border population has better health than it did in 1985 because falciparum malaria transmission is much reduced and access to health care improved once SMRU set up clinics. Nonetheless, the population remains at high-risk for infectious diseases and emerging drug resistance. This contributes to its poor health status, which, according to Investigator 02, is low when compared to the Thai population, whose life expectancy is 73.6 years [23]. The border population probably has a life expectancy of less than 65 years^i^, though there is no data to verify this. This is a sizeable gap in health status from the optimal level (84 years).

SMRU data indicates that the border population’s infant mortality rate is 50 per 1,000 live births. Comparatively, the infant mortality rates in Myanmar, Thailand, and Japan are 47, 15.9, and 2.2 per 1,000 live births respectively [23]^j^. The maternal mortality rate is estimated to be 79 per 100,000 live births in the refugee population and 252 per 100,000 live births in the migrant population [22]. (More than 75% of trial participants are from the migrant population.) The maternal mortality rates in Myanmar, Thailand, and Japan are 200, 48, and 5 per 100,000 live births respectively [23]^k^. The border population is then an acceptable host community under the framework, but, as its health continues to improve, SMRU may need to consider further expanding its research population.

#### ***Selection of the health condition***

Vivax is the main cause of malaria in the border population^l^. While falciparum has a greater impact on morbidity and mortality, vivax can cause significant morbidity. Unlike falciparum, the vivax parasite has liver stages that can remain dormant for weeks or months. These liver-stage parasites are not cleared by chloroquine (the standard of care on the Thai-Myanmar border), so each vivax infection is associated with multiple relapses [18]. Vivax can cause chronic anemia. In pregnancy, it is the most common cause of malaria-related anemia and low birthweight. The disease sometimes results in death. T-CAB member 04 confirms this, detailing her neighbor’s experience with the illness:

One of my neighbors, who were able to walk come-and-go, went to examine malaria virus and found out that there was none. He even hospitalized once at Koko Hospital, but wasn’t happy. So, he went to take the blood out this [SMRU] clinic and found virus and then found no virus at another time. He’s always sick and then has become healthy. A moment later, he was sick again. So, his children came back and took him to the Mae Tao Hospital. Three or four days later, he couldn’t live anymore and died finally. He died although he looked normal. That’s because the malaria virus got into liver.

While the incidence of vivax is high and relapses can result in chronic anemia, it typically does not cause severe illness in adults. Trial investigators do not consider it to be the top health concern of the border population. Investigator 01 states that

[v]ivax is a problem but not a serious—I would not put that as the number one priority in terms of health in the population. It probably goes after respiratory infection, diarrheal disease, and in terms of public health, tuberculosis is emerging as a big problem. Of course, if you look at just the sheer numbers, of course, we still treat many more cases of vivax than we treat tuberculosis, but it’s difficult to compare because one is a disease that almost you would, you know, could compare as a flu, as a mild flu, except that in young children, in very young babies, then it can be dangerous and in pregnant women it’s not very good, but in adults it’s like a flu, so you take three tablets of something and then you are done.

While vivax can have serious consequences for children and pregnant women, malaria is no longer the main cause of severe disease in the border population. Other infectious diseases are becoming more important—respiratory illnesses, diarrheal diseases, TB, and, within the TB population, 20% are HIV positive [18].

Even so, T-CAB members and VHX trial participants acknowledge that malaria continues to be a concern. Despite the reduced falciparum burden, many participants said they are scared of dying from cerebral malaria (falciparum) during interview [18]. For T-CAB members and trial participants, common reasons for being worried about vivax are that the disease will cause death or affect their capacity to work (see Table 1). T-CAB members and trial participants also identified non-malarial illnesses as being of concern, including cancer, dengue, and TB (see Table 2).

**Table 1:** T-CAB members and VHX trial participants’ views on whether malaria is a health concern

***Interviewee***	Identifies malaria as a health concern*	***Reason(s)***
T-CAB Member 01	No.	Has gained knowledge on how to prevent malaria.
T-CAB Member 02	Yes.	If gets malaria, cannot work. If gets cerebral malaria, can die.
T-CAB Member 03	Yes. Thinks people in his village worry as well.	If gets malaria, cannot work. “I cannot eat foods, I will not be able to do any kind of social activities and family matter and then it can effect to all of our business sector, social sector, and education sector.” If severe, malaria virus can cause death.
T-CAB Member 04	Yes. Thinks people in her village worry as well.	Large (falciparum) and small (vivax) malaria virus can cause death.
Trial Participant 01	Yes. And worries about malaria repeating again.	If unhealthy, cannot work.
Trial Participant 02	Yes.	Has seen people die from malaria.
Trial Participant 03	Yes, worries most about malaria virus.	Scared of “losing my physical body”.
Trial Participant 04	Yes, worries most about malaria virus.	Can die if don’t receive treatment.
Trial Participant 05	Yes, worries for cerebral malaria.	Can put life in danger.
Trial Participant 06	Yes.	Scared of dying, forgetting things, going crazy, becoming unconscious.
Trial Participant 07	Yes, worries for cerebral malaria.	If virus goes to brain, can die.
Trial Participant 08	Yes, worries for cerebral malaria.	Die if virus goes to the brain.

*Note: T-CAB members are specifically referring to vivax when they say they are worried about malaria, as they were asked “Do you worry about getting vivax malaria?”. However, trial participants were asked “Do you worry about getting malaria?” because they were unlikely to know that there are different types of malaria. As a result, their answers may refer to falciparum or vivax. Where trial participants mention cerebral malaria or its symptoms (e.g., trial participants 05–08), they are expressing a concern for falciparum. Trial participants 02–04 may also be referring to falciparum rather than vivax because falciparum is more commonly associated with death than vivax. Of the trial participants interviewed, trial participant 01 is the most likely to be identifying vivax as a concern, as he mentions relapsing.

**Table 2:** T-CAB members and trial participants’ non-malarial health concerns

***Interviewee***	***Identified non-malarial health concerns***
T-CAB Member 01	Cancer
T-CAB Member 02	TB and cancer. He is as concerned about these two illnesses as he is for malaria.
T-CAB Member 03	Diarrhea and dengue
T-CAB Member 04	TB and cancer
Trial participant 01	He is scared of getting diseases other than malaria and mentions “diseases of the stomach”, which may refer to gastroenteritis.
Trial participant 02	She does not worry about other diseases. “As I have never had it before, I do not worry about it.”
Trial participant 03	Abdominal disease
Trial participant 05	Dengue and HIV/AIDS
Trial participant 08	He is only scared of sexually transmitted diseases (and malaria).

Although vivax may not be the top health priority of the border population, it would be inaccurate to characterize SMRU’s focus on malaria as a deviation from framework requirements. When SMRU was established, falciparum was the main health problem of the border population^m^. Successful implementation of SMRU’s research results has meant that falciparum is of lesser priority now. Where the burden of disease in a host community changes significantly over time, ‘research for health justice’ obliges long-term collaborations to modify their research practice to reflect those changes. SMRU is now broadening its research agenda to include respiratory infections, non-malarial fever-related illnesses (dengue, leptospirosis, scrub typhus), maternal and child health, and TB. The decreasing burden of falciparum has led SMRU to diversify its research agenda. The VHX trial demonstrates that an immediate shift in research focus to new health priorities may not be possible.

Thus, the selection of vivax as the condition-under-study may not meet framework requirements if the border population’s current burden of disease is considered alone. When the context of SMRU’s broadening research agenda and history is taken into account, the disease target can be said to reflect the shift in SMRU’s research agenda in accordance with the changing pattern of disease on the border. Vivax now causes reasonably substantial morbidity given its relapse rate and incidence. Vivax is enough of a concern to warrant being a focus of SMRU’s research as it transitions the focus of its research agenda, provided there is a need for clinical research on the disease. Even so, its selection as a research target draws attention to the fact that SMRU has not yet been able to move the focus of the majority of its research to diseases of higher priority such as respiratory infections, diarrheal diseases, or TB. These newer research targets (non-malarial illnesses) comprise a minority of the studies currently being performed by SMRU.

A need for research on vivax exists because, although chloroquine treatment for vivax on the border is moderately effective, there is anecdotal evidence that resistance is emerging. This must be investigated. Given chloroquine does not clear liver-stage parasites, there is a further need to determine whether another existing treatment for vivax (primaquine) can be optimized and made safe for use by the border population. WHO and Thailand’s Ministry of Public Health currently recommend primaquine as the first-line treatment for vivax because it can clear liver-stage parasites. Even so, this recommendation tends not to be implemented by medical NGOs on the Thai-Myanmar border. As confirmed by Investigator 05,

primaquine has been quite widely recommended, but I can’t think of another drug where there’s a greater disparity between the recommendation and the use… And the reason for that is that primaquine is potentially dangerous. People who have a genetic abnormality, glucose-6-phosphate-dehydrogenase (G6PD) deficiency, it’s an X-linked genetic condition, which is found very widely in areas where there is malaria because it actually provides some protection against malaria. Now if you give people with this deficiency [primaquine], then they may hemolyze, so their red blood cells would blow up and this can be dangerous. So what we think happens is that doctors, clinics, and so on in the public sector have the recommendations but they also know the risks and they think ‘well, as I cannot test for this condition’… so they say ‘well, better be on the safe side, let’s not give it [primaquine]’.

G6PD deficiency is thought to be widespread in Asian populations. Along the Thai-Myanmar border, the NGOs who take responsibility for health care provision do not test for G6PD deficiency and, as a result, do not treat cases of vivax with primaquine. Though there is no formal evidence, the risks of deployment without routine G6PD testing or adequate hospital support are considered to outweigh the benefits.

Since primaquine isn’t routinely used, SMRU has not been able to reduce the burden of vivax in the border population. According to Investigator 01,

because we don’t use it [primaquine], it’s impossible to control vivax… So now that falciparum has been really suppressed, we started to think okay, let’s look again at vivax and at this drug and whether we can learn anything by utilizing new techniques and new assays at our disposal and see whether we could make it safer.

As such, the VHX trial is one of a series of studies being conducted by SMRU to optimize the use of primaquine for the border population.

Finally, for the selection of vivax to be consistent with the ‘research for health justice’ framework, the intervention-under-study must be appropriate (acceptable and implementable) for the host community. Primaquine is already used in SMRU clinics, though not routinely, and has been shown to be acceptable. Treatment with primaquine is also likely to be implementable at SMRU clinics. The series of studies on primaquine includes a comparative evaluation of current rapid diagnostic tests for G6PD deficiency. The intention is to do regular G6PD testing at SMRU clinics after the most effective test is identified.

#### ***Facilitating factors***

The VHX trial host community, disease target, and intervention-under-study are largely consistent with framework criteria. This is because of investigators’ commitment to performing research addressing the border population’s needs. SMRU investigators aim to study the most optimal and appropriate strategies to prevent and treat the main health problems of its research population. This requires deep comprehension of the illnesses that the border population experiences, which is why investigators have embedded SMRU in its host community. According to a VHX trial investigator,

[t]hat distance from the lab to the field is all the difference with SMRU. That we are in the field all the time… You have to be in the middle with the population. Work and most of your staff has to be people from the population, so your technicians, your computer staff, your nurses, they have to be from the population where you work. That’s sort of, you know, the close link is what makes it successful.

Most staff are recruited from the border population and trained to do research and provide clinical care. Staff not from the Thai-Myanmar border work alongside local staff. Together they measure the health needs of the population using epidemiological surveys, prospective cohorts, and clinic data collection systems [18]. Collectively, these methods give SMRU a strong understanding of the health-related burden of disease experienced by its research population.

#### ***Obstructive factors***

Despite SMRU investigators’ commitment to improving the border population’s health, the VHX trial focuses on vivax rather than a more significant contributor to its poor health status. Three factors explain why vivax and primaquine were selected as the focus of the trial: SMRU investigators’ commitment to performing studies of global and local significance, the tendency of researchers to specialize on specific diseases, and the research funding environment. Each factor makes it extremely hard for a long-term research collaboration to shift focus in accordance with a changing burden of illness in its host community.

SMRU investigators are committed to conducting research of direct benefit not only to the border population but also to populations living in malaria-endemic areas elsewhere [24]. When asked why the VHX trial focuses on vivax when the disease isn’t a large concern in the community, Investigator 01 responded:

Well, because we have been working on malaria for twenty years, so malaria is our main interest of research. So we have accumulated the experience and the know-how and the brain power with the scientists to work on malaria, so I think… it makes sense to because we are probably one of the few research centers on malaria in the world that can do this kind of studies, so if we don’t do these studies, there are very few other groups that do it. So I expect that the impact on the [border] population’s overall health is going to be small because the problem is not that big but when you are going to utilize the finding elsewhere, then you can multiply the impact. Like we did for the treatment of falciparum malaria… For vivax, we could argue that it’s not the top priority anymore in the population, except that by studying vivax here we could demonstrate that the parasite is becoming more resistant to the main treatment, which is chloroquine, and, therefore, it’s even more important to suppress it and control it because if we don’t then the resistance is going to spread further. Once the resistance has spread and increased, then the impact on the population is very significant because you have to change your treatment and your drugs.

Investigator 01 describes the potential for the VHX trial to have local and global impact. Locally, the research may lead to better treatment for a common disease in the face of emerging resistance. From a global perspective, the research is important because, if chloroquine resistance has emerged, controlling vivax in the border population is necessary to diminish the likelihood of resistance spreading. Findings regarding the safe, effective use of primaquine can also be applied beyond the Thai-Myanmar border to other Asian populations. This will advance the goal of global malaria elimination.

Funding for the trial was sought from Wellcome Trust as part of a larger Programme Grant on primaquine. The Trust’s Programme Grant scheme (which no longer exists) funded biomedical research into a series of related questions around a single topic (such as the use of primaquine) for a duration of five years. The scheme did not give strong consideration as to whether proposed studies focused on local health concerns or would be performed with worst-off communities. The four key criteria upon which Programme Grant applications were assessed were the science, the significance of the research question, value for money, and investigators’ track records. Of these criteria, the first was the most critical. As affirmed by Wellcome Trust representative 01,

the Programme Grant mainly is a scientific decision… in terms of: is this good science, what is the research question, why is the research question important, will this project answer that research question, is the project designed in an appropriate way, could it be improved, does it need improvement and if it needs improvement, that would generally suggest that this is not a good proposal.

The *global* significance of research questions may have been a factor when judging their importance and value for money. Wellcome Trust representative 01 stated that if a proposal is

important to the local population and has translatable outcomes that would be appropriate to different populations in other areas, then obviously that’s a higher priority than only has, you know, the outcome is only applicable to a small group of people in one region of the world… And things that have translatable outcomes, so that larger numbers of people, will have the bigger chance of success… The more people it will help or improve the quality of life, the better investment you’re making.

This suggests that research questions whose answers are useful on a broad scale may be ranked higher, provided proposed studies have a solid scientific design and investigators have a good track record. Local relevance is not really a consideration beyond the fact that the disease-under-study is prevalent in the host community.

Wellcome Trust’s position statement on research involving people in developing countries does require the conduct of research whose outputs can “become available to patients in those areas and be deliverable within existing structures (or structures which are to be or could readily be developed)” [25]. Nonetheless, the review process for Programme Grant proposals did not explicitly reward proposals for testing “locally applicable” interventions [25]. According to Trust representative 02, considering whether outcomes of a study can be implemented where the research will be conducted is “not the main role of the funding committee”. He also notes it is often beyond the scope of the funding committee to assess projects’ chances of local applicability.

Wellcome Trust external reviewers and funding committees did not rely on criteria similar to those of the ‘research for health justice’ framework to evaluate Programme Grant applications. The Primaquine Programme Grant (of which the VHX trial is part) was funded because it was rated highly in terms of its science, research question, and investigators’ track records^n^. That it promotes the health of the worst-off reflects the commitment of investigators rather than Wellcome Trust’s funding scheme.

Ultimately, SMRU investigators’ expertise in malaria research and Wellcome Trust’s emphasis on track record and value for money help explain why the VHX trial does not focus on a higher priority illness in the border population. Despite these obstacles to remaining responsive to significant local health problems, SMRU *is* beginning to broaden its research agenda, but Investigator 05 confirms that SMRU will keep a focus on malaria. He states that

we’re not going to move too much though. I think that, you know, we’ll be building on strengths rather than completely new areas. So I think it’s a reasonable chance of success.

SMRU can only diversify so far and continue to be successful in its grant applications and conduct of high-quality research.

#### ***Fulfillment of obligations***

Oxford University and VHX trial investigators’ selection of a research target is generally consistent with framework obligations, but Wellcome Trust falls short of its obligation to fund ‘research for health justice’. Wellcome Trust allocated US $65 million to research on neglected diseases of the developing world in 2009 [26]. Here, neglected diseases are defined as diseases that are “overwhelmingly or exclusively incident in the developing countries” and diseases that are “incident in both rich and poor countries, but with a substantial proportion of the cases in the poor countries” and where R&D is “not in proportion to global need or addressed to the specific disease conditions of poor countries” [27]. US $65 million does not comprise even 10% of Wellcome Trust’s annual £600 million (US $967 million) health research expenditure, though this excludes funding for research capacity strengthening in LMICs [28]. We suggest that the Trust’s investment in research in LMICs could increase.

Wellcome Trust’s funding could also be better targeted to projects that are more consistent with framework requirements. This will entail modifying existing grants programs. In 2011, the Trust began awarding long-term funding to individual researchers rather than to projects and programs. Its New and Senior Investigator Awards provide five to seven years of funding for investigators whose research focuses on important scientific questions [29]. To advance ‘research for health justice’, Wellcome Trust might consider giving preference to applicants whose clinical research

• tackles scientific questions relating to diseases causing significant morbidity and mortality in LMIC host communities,

• involves working with worst-off communities,

• builds long-term relationships with and the capacity of LMIC researchers, and

• will generate outputs that can be used in host communities.

### Research capacity strengthening

For SMRU’s VHX trial to meet framework criteria for research capacity strengthening, it should be conducted as part of long-term partnerships with host country (Thailand) institutions and researchers from Myanmar, Thailand, and/or the border population. Such collaborations should gradually build LMIC researchers and institutions’ capacity to conduct clinical research independently. As part of this process, the VHX trial ought to involve the transfer of skills, knowledge, and resources to these researchers [8].

#### ***Research capacity strengthening during the VHX trial***

The VHX trial has strengthened the capacity of SMRU’s Karen and Myanmar clinic and laboratory staff to collect clinical research data on vivax [18]. All clinic staff involved in the trial received training on malaria and trial processes, including participant recruitment, case report form completion, and study sample collection. Staff were taught how to do malaria smears and hematocrits, which were essential to identifying key trial endpoints. For some clinic staff, this built research capacity, but for others it did not because they had been involved in earlier SMRU trials. Across the trial sites, research experience varied considerably prior to the trial commencing. According to Investigator 03,

[i]n Mawker Thai, none of them had [research experience]. Yeah, they’ve come a long way. They’ve come a long way. In Wang Pha, they had quite a lot of experience, so they were fine. In Mun Ru Chai, they’d had some experience. Mae Kon Ken a lot of experience. And Mae La, one medic has a lot of experience. She’s actually since gone and the others had little bit.

As most of SMRU’s malaria studies have been conducted at Wang Pha Clinic, its staff are experienced in data collection and patient care. For them, the VHX trial meant learning to use new machines such as the G6PD fluorescence machine rather than significantly enhancing their research skills.

The VHX trial has built capacity amongst Mawker Thai Clinic and Mae La Clinic medics, nurses, and home visitors. An investigator affirmed that this is because

since I started the VHX, we’ve lost a number of senior staff due to relocation, immigration, and then it’s required that we ask a lot out of the more junior staff. And I’m very pleased with the way some of them have risen to the challenge and been able to take on take on the leadership of the study.

Due to the resettlement of senior staff at Mawker Thai and Mae La clinics, junior staff took on senior roles for the VHX trial. The VHX trial has also built the research capacity of SMRU laboratory staff to use new techniques and conduct new assays.

VHX trial training was conducted in two stages. First, staff involved across all five trial sites were transported to SMRU’s Mae Sot office for a training day. Two local SMRU staff members were responsible for teaching staff how to carry out the trial’s consent process. A VHX trial investigator was responsible for explaining the purpose of the trial and its methods, processes, and endpoints. Second, the same investigator and two local staff members went from site to site to practice the recruitment process with the SMRU staff running the trial.

#### ***Alignment with ‘research for health justice’***

The framework has five requirements for research capacity strengthening at the project level. First, it calls for ICR to strengthen clinical research capacity on health conditions that are major contributors to poor health in worst-off communities. Although vivax may not be the top contributor to poor health on the Thai-Myanmar border, it causes reasonably significant morbidity. Second, ICR should strengthen clinical research capacity on conditions where such research is needed. As noted, a need for research on vivax exists because resistance to chloroquine may be emerging and treatment for vivax has yet to be optimized for the border population.

Third, ‘research for health justice’ demands that when high-income country researchers and institutions perform ICR in LMICs, it be conducted through partnerships with LMIC researchers and institutions. The VHX trial is conducted through the Mahidol-Oxford Tropical Medicine Research Unit, established in 1979. SMRU was created as a field unit of that collaboration. The trial is led by Oxford University investigators, with assistance from American and Myanmar investigators. No investigators are from the border population or Thailand. VHX trial investigators are, however, working with members of the border population to conduct the study. Although physicians from Myanmar and high-income countries fill more senior SMRU positions, individuals from the border population serve as clinic, laboratory, and research staff. The combination of Myanmar investigators and local research staff is the closest achievable approximation of a research group from the border population. While external investigators did not collaborate with Thai researchers, their partnering practice (with the border population and Myanmar doctors) is consistent with ‘research for health justice’.

Fourth, the framework requires that ICR partnerships be of lengthy duration so that they build the capacity of local partners to perform clinical research. The VHX trial is being conducted as part of a 25 year partnership between, at the institutional level, Mahidol and Oxford universities and, at the individual level, Oxford University researchers and members of the border population. Institutional capacity has been built at the Mahidol-Oxford Tropical Medicine Research Unit to undertake grants administration, ethics review, data monitoring, and logistics. The Clinical Trial Support Group is now responsible for these tasks. Its staff are mostly Thai, though its head is Malaysian. SMRU’s research capacity-building is mainly directed towards the border population and doctors from Myanmar and Western countries. The VHX trial, for example, supports the PhD training of an American doctor. However, ‘research for health justice’ emphasizes building the capacity of Myanmar and Thai physicians, wherever possible, rather than physicians from high-income countries.

SMRU has not built the border population’s independent clinical research capacity. After 25 years, researchers from Oxford University remain in-charge of SMRU and are responsible for making major research decisions. These investigators identified the research questions to be investigated as part of the Primaquine Programme Grant, chose to apply to Wellcome Trust for funding, set the VHX trial objectives, and determined how the trial was designed. The two Myanmar doctors who served as co-investigators on the VHX trial were not involved in these processes. Local staff are responsible for participant recruitment and data collection. Data analysis will also be done primarily by local staff from SMRU’s data management center, but the plan for data analysis will be designed by external investigators and the Mahidol-Oxford Tropical Medicine Research Unit’s statistician. VHX trial publications will be written primarily by investigators from Europe and the United States.

Finally, the framework calls for investigators to build research capacity at the individual level (i.e., of persons from Myanmar, Thailand, or the border population) during the VHX trial. Trial investigators have strengthened the capacity of SMRU staff from the border population to manage the day-to-day running of clinical research. This involved skill-building in participant recruitment, data collection, and leadership.

#### ***Facilitating and obstructive factors***

Successful research capacity strengthening at the individual level has been achieved due to Wellcome Trust funding practices and SMRU being embedded in its host community. Wellcome Trust representatives affirm that research capacity strengthening is one of the Trust’s “major missions”. Where research is undertaken in LMICs, the Trust “expects some capacity-building—training of local staff, encouragement of local staff to take part in the research, train them in PhDs, if possible” and “advocates that things are done as locally as possible to build capacity”. Accordingly, there was an allocation in the VHX trial budget for training and capacity-building related to the trial. As confirmed by Investigator 05,

there wasn’t a specific budget that was set aside for teaching that was not germane to the trial. There was certainly a budget for developing the trials, training people about the trial, you know, doing the G6PD testing, all that stuff… so it’s related to the trial in this budget.

Training provided by investigators then focused solely on the VHX trial and was directed almost exclusively to members of the border population. This is consistent with ‘research for health justice’ requirements at the project level.

Funding arrangements with Wellcome Trust have also made possible the long-term partnerships between Oxford and Mahidol universities and between SMRU investigators and the border population. The Mahidol-Oxford Tropical Medicine Research Unit and SMRU are part of Wellcome Trust’s Major Overseas Programme in Thailand, which has been funded since 1979, with the Trust providing financial resources to create facilities and infrastructure for research and to support fellowships, project grants, and program grants. Some of this funding can be used for research capacity strengthening.

SMRU’s practice falls short of ‘research for health justice’ with respect to building the ability of Karen and Myanmar individuals to perform clinical research independently. (Please note, this comment relates to the outcome of SMRU’s 25-year collaboration not the VHX trial, as single trials are not expected to develop such a high level of research capacity.) After 25 years, members of the border population are not involved in designing trials or applying for funding. Investigator 01 elaborates on why this is and why it is unlikely that local staff will ever run SMRU:

*I thought many years ago that it could be, but I have sort of given up the idea of building a 100*% *bridge Karen research organization. It’s not going to happen. And the reasons that I could identify are that they don’t have the skills. I mean, not the skills, I’m not talking about the technical skills, but they don’t have the educational background and so even if you tried to you know train and give training, they are lacking all the basics and so you have to start from zero… Plus, it has happened over the 20 years we had some people who were academically bright or some students in Myanmar who came over here and they could have developed their own research ideas, but they went or are resettled in America, in Australia, and so this turnover means that we can’t keep people long enough to develop their skills up to a level where they become, except maybe the team that does data entry, the data management people, they now are quite independent and can develop their own databases, their own algorithms for cleaning up the database.*

Educational opportunities are limited in Myanmar, particularly for the Karen. They face considerable persecution from the military and are repeatedly forced to flee their homes and resettle within Myanmar. This is not conducive to gaining an education. Irrespective of their lack of education, some clinic staff could one day assume a leadership role at SMRU. However, the resettlement process for stateless persons and the mobility of the border population mean that such individuals do not remain at SMRU for long. Even lower level research roles must be re-assigned regularly due to resettlement and migration. It, therefore, may not be reasonable to expect research capacity to be built to a level of independent proficiency when working with stateless populations who lack access to secondary and tertiary education opportunities.

In recent years, SMRU has recruited six Myanmar physicians, two of whom are doing a PhD. In other SMRU trials, these doctors have assumed the role of investigator primarily responsible for trial management. As a longstanding collaboration, this commitment to training investigators from Myanmar is integral to ‘research for health justice’. However, the VHX trial is supporting the PhD training of an American doctor rather than an individual from Thailand or Myanmar. The fact that PhD students are not drawn from the border population is unsurprising for the aforementioned reasons. According to Investigator 01, SMRU does not train Thai doctors because they are not interested in its research. Thai doctors are not willing to come and work in Mae Sot because it is viewed as too remote and dangerous. Historically, there is animosity between the Thai and Myanmar populations. SMRU trains more Western doctors for PhDs than Myanmar doctors because there are few candidates from Myanmar. In part, this reflects the lack of educational opportunities and the fact that the job involves working in a conflict zone that is particularly dangerous for the Karen and others from Myanmar. These factors stymie external investigators’ efforts to develop local investigators’ (from Myanmar) capacity to take over SMRU’s leadership.

#### ***Fulfillment of obligations***

Through its Major Overseas Programme in Thailand, Wellcome Trust upholds its obligation to create funding schemes that support long-term research collaborations between high-income country and LMIC institutions. The scheme is partly responsible for financing a collaboration between Oxford researchers and research staff from the border population.

Oxford University behaved consistently with framework requirements by establishing a long-term collaboration with Mahidol University and strengthening its capacity to administer grants and coordinate ethics approvals for clinical trials. Oxford researchers uphold their obligation to set up collaborations with researchers from LMICs by performing trials with doctors from Myanmar and staff from the border population. As part of the VHX trial, they have built the research capacity of individuals from the border population, which is consistent with framework demands. Over the years, for reasons that are understandable, external investigators have not built their local counterparts' capacity to conduct clinical research independently.

### Post-trial commitments

This section assesses the likelihood that vivax malaria treatment practice will change in the host community post-trial. The framework requires these changes be financed by a global health institution and be delivered to trial participants and their communities though the Thai health system.

#### ***Likely changes to treatment practice associated with VHX trial***

Depending on the VHX trial results, investigators affirm that any one of three post-trial scenarios is possible, with the first two being most likely:

1. The VHX trial confirms that chloroquine resistance on the border has reached unacceptable levels. It also shows that primaquine is the most effective treatment for vivax. The local treatment is changed to primaquine directly after the VHX trial. This primaquine regimen is later amended based on the results of the next trial funded under the Primaquine Programme Grant, which tests different primaquine regimens.

2. The VHX trial shows that chloroquine resistance on the border has not risen to unacceptable levels. It also shows that primaquine is the most effective treatment for vivax. The local treatment for vivax is not changed until after the trial testing different primaquine regimens is completed.

3. The VHX trial shows that chloroquine resistance on the border has not risen to unacceptable levels. It also shows that chloroquine is the most effective treatment for vivax. There is no change in the treatment for vivax.

If the second scenario eventuates, the significance of the VHX trial will be scientific, as it will have improved the design of the subsequent primaquine regimen study. Data from the VHX trial suggests that artesunate is ineffective in treating vivax relative to chloroquine and primaquine because patients treated with it relapse more frequently. As a result, the primaquine regimen study will evaluate seven and fourteen-day regimens of primaquine with DHA-piperaquine rather than artesunate, as was originally intended.

The high likelihood that SMRU will change the treatment regimen for vivax in its clinics after the VHX trial is reiterated by T-CAB members. When asked to comment on the benefits of SMRU research, they noted that the medicines tested by SMRU will assist and treat “future people” and “future generations” of their communities. T-CAB member 02 was asked “so, regarding that research on this mosquito virus, what does the research benefit for this clinic?” In response, he states: “this new medicine is for this clinic in upcoming one day. If the old medicine is not effective any more, then this new medicine benefits for this clinic”. Where drug resistance develops, SMRU clinics can make available treatments proven efficacious by its research. T-CAB members recognize that once SMRU research indicates that new treatments are more effective than those currently in use in its clinics, SMRU doctors and medics will prescribe the new medicines.

Ultimately, our data suggest that changes to treatment practice in the VHX trial host community will take place if needed. However, if high levels of chloroquine resistance are not evidenced by the VHX trial, the obligation will only be fulfilled *after* the primaquine regimen study is completed.

#### ***Implementing changes in local treatment practice***

Since SMRU runs clinics on the border, translating new evidence into local treatment practice is straightforward. As affirmed by Investigator 01, “because we work with the population and in the population, from tomorrow, we decide to change the treatment, we do it”. When researchers are confident in trial findings, changes are implemented in clinical practice. To do so, the Malaria Handout (i.e., treatment guidelines for malaria written by SMRU) is revised [18]. The process begins with a meeting of SMRU doctors to discuss

what the changes are, why we have to change, what the evidence is, and we discuss the practicalities, we discuss the cost, we discuss you know all the implications, then we change our guideline and then we have a phase where we train the staff to adapt to the changes and then they start implementing the changes in the clinics.

Once changes to the Malaria Handout have been discussed, different doctors are given sections of the document to revise. For the next round of revisions, the vivax portion will probably be allocated to the American investigator in charge of managing the VHX trial. Revisions to the Malaria Handout on the treatment regimen for vivax will be based on the results of the VHX trial and subsequent primaquine regimen study. These changes will be finalized by the head of SMRU, Francois Nosten. The doctor-in-charge at each SMRU clinic will be responsible for educating staff on the new treatment practice. These clinics are utilized by trial participants and their communities.

After SMRU revises the Malaria Handout, it is shared with the five medical NGOs operating on the Thai-Myanmar border. It, thus, affects treatment practice in clinics utilized by the wider border population. To inform the NGOs about the new treatment guidelines, SMRU hosts an annual workshop where SMRU doctors distribute the revised Malaria Handout and give training on the implementation of new procedures [18]. Whether the NGOs decide to change their policy is up to them, but SMRU’s track record and the strength of evidence it provides means that the NGOs usually adopt the Malaria Handout as their treatment guidelines.

By changing the treatment regimen for vivax in SMRU clinics, investigators will uphold the obligation to change treatment practice in the VHX trial host community. This goes beyond what is expected by ‘research for health justice’. The framework does not oblige *external trial investigators* to provide post-trial benefits because it falls outside the scope of their typical role. It recommends post-trial changes to treatment practice be coordinated by a global health institution and implemented through local health systems or through medical NGOs affiliated with them. Researchers are only responsible for identifying and supporting a local health structure(s) to apply for funding from a global health institution. The Thai health system has not taken responsibility for providing health care to the border population (aside from legal migrants who can afford health insurance) and is largely inaccessible to them. Although SMRU investigators will deviate from framework requirements by implementing changes in treatment practice through a non-state system, they cannot be held responsible for decisions of the Thai government. They deserve praise for meeting the responsibilities of others.

#### ***Facilitating factors***

The factors facilitating implementation of changes to local treatment practice for vivax include SMRU’s dual role as a research unit and health care provider; the existence of the Global Fund to Fight AIDS, Tuberculosis, and Malaria; primaquine being off-patent; and SMRU clinics’ position outside the Thai health system. By assuming a dual responsibility, SMRU enables the efficient and effective translation of its research results into practice in clinics used by its research population [18]. SMRU will be able to forecast how much primaquine it needs, procure it, and deliver it to patients from the border population.

Procurement and delivery of primaquine will probably be financed by the Global Fund, a global health partnership that supports scaling-up access to proven interventions for HIV/AIDS, TB, and malaria. As it is a health care provider, SMRU can obtain funding through the Global Fund and is not solely reliant on research funders. (Most research funders, including Wellcome Trust, do not grant funding for post-trial activities.) Primaquine is an older drug, so it is off-patent and inexpensive. Numerous manufacturers exist and, with Global Fund assistance, cost will not prevent SMRU from buying enough primaquine to meet the border population’s need. Primaquine will be free-of-charge at SMRU clinics, ensuring affordability at the patient-level.

SMRU can change treatment practice in its clinics as it chooses. It does not have to follow Thai treatment policy because it does not treat Thai people. Availability, affordability, and adoption of the new primaquine regimen in the host population can, thus, each be achieved.

#### ***Obstructive factors***

Thailand’s unwillingness to fully open its health system to stateless populations would, in most cases, be a significant obstacle to external actors upholding their post-trial benefit obligations. SMRU has overcome this by establishing a health care system for the border population that supplements the Thai system. Although ‘research for health justice’ does not oblige external research actors to improve health care systems in host communities of ICR, assuming a dual role as research unit and health care provider is a pragmatic response in the context of working with stateless populations for whom Thailand and Myanmar are unwilling to uphold their obligations. Nevertheless, since SMRU clinics are not part of a state-run health system, this strategy raises the issue of long-term sustainability of treatment access. For example, a problem may be caused by the eventual retirement of SMRU head, Francois Nosten.

#### ***Fulfillment of obligations***

SMRU investigators are high likely to change treatment practice for vivax on the border after the primaquine regimen study is completed. The Global Fund will finance the provision of the new primaquine regimen to the host community.

## Conclusions

This paper provides further evidence of how empirical research can be employed as a useful supplement to conceptual research. Our study of the VHX trial revealed shortcomings of the ‘research for health justice’ framework and identified limits to what it may be reasonable for the framework to require in the context of research with stateless populations.

The SMRU research model is worthy of replication. The VHX trial is only minimally inconsistent with ‘research for health justice’ requirements in three areas, which, given that this was a retrospective analysis of a new ethical standard, is quite remarkable. The selection of vivax as the health condition-under-study indicates that it may not be possible for long-term collaborations to alter their research agendas to align with changes in the burden of disease in their research populations in the short to medium term. Instead, they may have to make the transition much more gradually due to the disease-specific nature of research expertise and the research funding environment.

After 25 years of collaboration, the border population’s research training does not cover the capacities necessary for conducting research independently. Karen and Myanmar refugees, migrants, and displaced persons are quite mobile, creating regular staff turnover, and lack access to a secondary and tertiary education system, making it largely impossible to train them to become investigators. This suggests that the framework may require too much of long-term collaborations in terms of research capacity strengthening for stateless populations. It also highlights the fact that capacity-building obligations need clear boundaries in the same way as obligations to provide ancillary care and treatment post-trial. The content and extent of capacity-building obligations need to be clarified (i.e. how much and what sort of capacity-building is required in different contexts and for different sorts of people).

Post-trial benefits will not be provided through a state-run health care system after the VHX trial. The case study highlights the reality that many LMICs do not make health care accessible to refugee and migrant populations within their borders. The VHX trial demonstrates that framework requirements are achievable rather than unrealistic and aspirational in such contexts. It shows that researchers can uphold obligations linking ICR to justice in global health, despite two LMICs failing to fulfill their duty of care to the border population, and describes their successful strategy for doing so. By embedding their institution in its host community, obtaining money from a funder committed to long-term research capacity strengthening, and securing financing for post-trial provision of medicines from the Global Fund, SMRU investigators have been able to adhere to ‘research for health justice’ and will make benefits available to the host community post-trial.

While this case study shows that external research actors from high-income countries can uphold ethical obligations linking ICR to justice in global health and offers an initial description of how they might do so, other successful strategies still need to be identified, as LMIC settings differ considerably. The context of the VHX trial is somewhat uncommon, as it involves refugee and migrant populations who cannot access state health systems. More frequently, ICR is performed with populations that are citizens of specific states and in settings where state health systems are accessible but under-resourced and offer limited care. Additional empirical research is needed to determine what approaches are relied on in these settings to link trials to justice in global health.

## Endnotes

^a^All references to the worst-off refer to those who are worst-off *in terms of their health*.

^b^The 2013 *Declaration of Helsinki* gives the primary obligation for the provision of post-trial benefits to researchers, sponsors, and host country governments in Paragraph 34. The 2002 CIOMS guidelines state that “the sponsor and the investigator must make every effort to ensure that any intervention or product developed, or knowledge generated, will be made reasonably available” in Guideline 10. Although other actors are mentioned in the commentary to Guideline 10, they are only tasked with being part of discussions and negotiations regarding making successful interventions available and distributed post-trial, rather than being assigned the responsibility of making them available.

^c^Please note that this paper is the latest paper in a series of conceptual and empirical papers on linking international clinical research to global justice. The other papers in the series are (in the order that they should be read): 1) Pratt et al. [14]. “Evaluating the capacity of theories of justice to serve as a justice framework for international clinical research.” *American Journal of Bioethics* 12(11):30-41; 2) Pratt, B. & Loff, B. [10]. “A framework to link international clinical research to the promotion of justice in global health.” *Bioethics*, doi: 10.1111/bioe.12009; 3) Pratt et al. [30]. “Ancillary care: Theory and practice in international clinical research.” *Public Health Ethics*, doi: 10.1093/phe/pht015; and 4) Pratt et al. [18]. “Closing the translation gap for justice requirements in international research.” *Journal of Medical Ethics*. doi:10.1136/medethics-2011-100301.

^d^The VHX trial was not designed to meet framework requirements.

^e^We do not know the legal status of all members of the border population with whom SMRU works. It likely varies from person to person. Although all Karen and Myanmar refugees, migrants, and displaced persons may not be owed formal legal obligations by Thailand, we take the position that Thailand has a *moral* obligation to provide health care to the border population.

^f^See Pratt, B., Lwin, K.M., Zion, D., Nosten, F., Loff. B, & Cheah, P.Y. (2013). “Exploitation and community engagement: Can CABs successfully assume a role minimising exploitation in international research?” *Developing World Bioethics*, doi:10.1111/dewb.12031.

^g^The aim of capacity-building under the framework is to support the stateless population without assisting or enabling the unjust government that it has fled.

^h^Registered economic migrants can access Thai health insurance for 1,300 baht per year. This insurance does not cover some conditions, including difficulties during pregnancy, dialysis, and antiretroviral treatment beyond that required to prevent mother-to-child transmission of HIV. Those refugees, illegal migrants, or displaced persons who manage to surmount significant physical and military barriers to reach Thai hospitals will also be treated. Getting to Thai hospitals requires overcoming obstacles such as leaving Mae La refugee camp, traveling twenty kilometres (or more) beyond the border into Thailand, and/or passing through military checkpoints intended to prevent refugees and illegal migrants from crossing into Thailand. Such barriers are not frequently overcome, but whenever a non-Thai patient, whether legally in Thailand or not, manages to present him/herself to the public hospitals, s/he is provided with care. If the patient is too poor to pay, this care is free.

^i^Life expectancy in Myanmar is 65 years [23].

^j^A country with an infant mortality rate of 50 per 1,000 live births would fall 175th out of the 222 countries listed in the CIA World Factbook.

^k^A country with a maternal mortality rate of 252 per 100,000 live births would fall 139th out of the 183 countries listed in the CIA World Factbook.

^l^The burden of falciparum malaria has fallen dramatically (99% in twenty years) in refugee camps and in villages in the border districts as a result of a control strategy designed by SMRU and implemented by all medical NGOs in the area.

^m^In 1985, malaria was the first cause of morbidity and mortality, accounting for 16% of deaths and 40% of MSF clinic consultations.

^n^Wellcome Trust representatives confirm the Primaquine Programme Grant application was successful because scientifically it was “a strong study in itself” and asked a compelling research question. SMRU investigators have a strong track record in the field of malaria research. External reviewers noted that the applicants were leaders in the field and uniquely positioned to carry out the studies.

## Abbreviations

CAB: Community advisory board; G6PD: Glucose-6-phosphate-dehydrogenase; ICR: International clinical research; LMIC: Low- and middle-income country; MSF: Médecins Sans Frontières; NGO: Nongovernmental organization; SMRU: Shoklo malaria research unit; T-CAB: Tak province border community ethics advisory board; VHX: Vivax malaria treatment; WHO: World Health Organization.

## Competing interests

Three of the co-authors of this paper are employed by SMRU. However, their contribution to data collection and the drafting of the paper did not result in the exclusion of any negative findings.

## Authors’ contributions

BP participated in the study design, data collection, and data analysis. DZ and BL participated in the study design and data analysis. PYC, KML, and FN participated in the collection of data. BP wrote the first draft of the paper and the co-authors contributed to the development of the final version. All authors read and approved the final manuscript.

## Pre-publication history

The pre-publication history for this paper can be accessed here:

http://www.biomedcentral.com/1472-6939/15/49/prepub

## Supplementary Material

Additional file 1 **Main requirements of the ‘research for health justice’ framework for international clinical trials.** The table identifies the criteria that single international clinical trials must meet in three areas (selecting a research target, research capacity strengthening, and post-trial benefits) in order to be consistent with the ‘research for health justice’ framework. Click here for file

Additional file 2 **Obligations under the ‘research for health justice’ framework.** The table identifies the obligations of justice of various types of external research actors in relation to three aspects of international clinical research (selecting a research target, research capacity strengthening, and post-trial benefits). Click here for file
