# Supplementary material for: Linking international clinical research with stateless populations to justice in global health
Source: BMC Med Ethics. 2014 Jun 26;15:49. doi: 10.1186/1472-6939-15-49 (PMC4085396; doi:10.1186/1472-6939-15-49)
Supplement: Additional file 1 — Main requirements of the ‘research for health justice’ framework for international clinical trials. The table identifies the criteria that single international clinical trials must meet in three areas (selecting a research target, research capacity strengthening, and post-trial benefits) in order to be consistent with the ‘research for health justice’ framework. [file 1472-6939-15-49-S1.docx]

Additional file 1: Main requirements of the ‘research for health justice’ framework for international clinical trials

| *Selection of the research target*   1. Host communities must meet the following criterion:    - Their populations exhibit a large gap in health status from the optimal level as demonstrated by surveillance data. For example, having a life expectancy of 65 years or less. The optimal level is 90 years. 2. The health condition and intervention-under-study must meet each of the following criteria:    - The health condition is a major contributor to host communities’ gap in health status from the optimal level.    - A need for clinical research on the health condition exists in host communities. This occurs when one or more of the following conditions is met:      - no effective prevention intervention or treatment exists for the health condition,      - existing treatments are losing effectiveness in host communities due to emerging patterns of resistance or other biological factors (of the pathogen, vector, or host),      - existing treatments are not effective in host communities because the illness sub-type has a large impact on treatment efficacy and no treatments have been developed that target the illness subtypes present in host communities,      - existing prevention interventions or treatments are not effective in host communities because they have not been adapted for resource-poor settings, or      - existing prevention interventions or treatments need to be optimised for a particular population.    - The intervention being tested is appropriate (acceptable and implementable) for use by participants and host communities. 3. Longstanding research collaborations should shift their research agenda to reflect any substantial changes in their host communities’ burden of disease over time. |
| --- |
| *Research capacity strengthening*  At the institutional and project levels research capacity strengthening should:   - Relate to a health condition that is a major contributor to shortfall inequalities in health in the community where ICR is undertaken. (Necessary) - Build research capacity on a health condition where clinical research is needed. (Necessary) - Be conducted through partnerships with LMIC research groups and institutions. (Necessary) - Involve the transfer of skills, knowledge, and resources that build the independent capacity of LMIC institutions and researchers to perform clinical research. (Necessary) - Generally be of lengthy duration and span more than one clinical trial. (Highly desirable) |
| *Post-trial benefits*  The provision of post-trial benefits should meet the following criteria:   - Consist of making successful interventions or treatment regimens sustainably accessible in host communities of ICR post-trial. - Be financed and/or coordinated by a (newly created) global health institution. - Be delivered through local (state-run) health systems wherever possible. |
