# Supplementary material for: Linking international clinical research with stateless populations to justice in global health
Source: BMC Med Ethics. 2014 Jun 26;15:49. doi: 10.1186/1472-6939-15-49 (PMC4085396; doi:10.1186/1472-6939-15-49)
Supplement: Additional file 2 — Obligations under the ‘research for health justice’ framework. The table identifies the obligations of justice of various types of external research actors in relation to three aspects of international clinical research (selecting a research target, research capacity strengthening, and post-trial benefits). [file 1472-6939-15-49-S2.doc]

Additional file 2: Obligations under the ‘research for health justice’ framework

| **Obligation-bearer** | **Obligations of justice** |
| --- | --- |
| National governments | Selection of research targets   - Create strong policy incentives for ICR that develops interventions to combat health conditions that are major contributors to worst-off communities’ shortfalls from the optimal level of health. - Abolish policies that create barriers or disincentives to performing this type of ICR.   Research capacity strengthening   - Create strong policy incentives for clinical research capacity strengthening in LMICs that is consistent with framework requirements. - Abolish policies that create barriers or disincentives to performing research capacity strengthening.   Post-trial benefits   - Enact legislation that requires an annual government contribution towards funding the mediating institution. - Abolish policies and legislation that prevent the use of research funding to provide post-trial benefits. |
| Funders | Selection of research targets   - Set up funding schemes for ICR that develops appropriate interventions to combat health conditions that are major contributors to worst-off communities’ shortfalls from the optimal level of health.   - Support surveillance work (for groups with long-term partnerships) and the option for subsequent implementation research as part of these funding schemes.   Research capacity strengthening   - Create funding schemes that support long-term collaborations between high-income and LMIC clinical research groups. - Create funding schemes that support long-term institutional partnerships to build LMIC institutions’ clinical research capacity.   Post-trial benefits   - Supply participants with interventions proven efficacious in phase II, III, or IV trials from the end of the trial proving efficacy until organisations obtain funding from the mediating institution to coordinate host communities’ access to the intervention. This obligation only applies where the local health system or NGOs can deliver the intervention and is for a fixed period agreed upon by the funder at the outset of all trials. |
| Sponsors | Selection of research targets   - Support researchers to design ICR partnerships and projects that develop appropriate interventions to combat health conditions that are major contributors to worst-off communities’ shortfalls from the optimal level of health.   Research capacity strengthening   - Set up long-term collaborations with institutions in LMICs (or at the regional level) to strengthen their clinical trial capacity.   - Build local counterparts capacity to administer grants and manage their research portfolios independently. (*Long-term relationship*)   Post-trial benefits   - Support international clinical researchers to liaise with local organisations to develop funding applications to submit to the mediating institution. |
| International clinical researchers | Selection of research targets   - Design ICR partnerships and projects that develop appropriate interventions to combat health conditions that are major contributors to worst-off communities’ shortfalls from the optimal level of health.   - Conduct surveillance with local research partners on host communities’ disease burden and provide the collected information to national and local governments. (*Long-term relationship*)   Research capacity strengthening   - Set up (long-term) collaborations with researchers and a research group(s) at a LMIC institution that builds their clinical research capacity.   - Build local counterparts capacity to conduct clinical research projects independently. (*Long-term relationship*)   Post-trial benefits   - Advocate for the creation of the mediating institution or the expansion of an existing institution to create access to successful interventions post-trial. - Alert the mediating institution of successful study results as soon as they are determined (i.e., prior to publication). - Support/partner with the local health system and/or NGOs to apply for funding from the mediating institution to create access to efficacious interventions in host communities. |
| Multilateral agencies and other global health institutions | Selection of research targets   - Improve the global health information system and build countries’ capacity such that they are able collect and report surveillance data on their worst-off populations’ burden of disease. (WHO and its regional offices)   Research capacity strengthening   - Assist LMIC governments to strengthen their national health research systems so that they have the financial, physical, and human resources to conduct clinical research on diseases driving their populations’ gap in health status from the optimal worldwide level. (WHO, with support from UN and COHRED)   Post-trial benefits   - Create and maintain a mediating institution charged with coordinating access to newly registered products in the host communities in which they were originally tested (phase II, III, IV trials). (Global health institutions and actors) |
